# My daughter knows over 21 people that have died from drug overdose or suicide: cross-sector rural provider perspectives on adverse childhood experiences

**DOI:** 10.3389/fpubh.2026.1891916

**Published:** 2026-10-28

**Authors:** Ashley E. Douglas, Julie M. Kapp

**Affiliations:** Department of Public Health, College of Health Sciences, University of Missouri, Columbia, MO, United States

**Keywords:** adverse childhood experiences, qualitative, rural, training, trauma-informed care, workforce

## Abstract

**Background:**

Cumulative exposure to adverse childhood experiences (ACEs) is associated with an increased risk of substance use, overdose, and suicide. Rural children are more likely to experience most ACEs. There is limited literature contextualizing rural individuals’ experiences with ACEs. Public Health 3.0 calls for multisector partner engagement to generate collective impact. The purpose of this study was to evaluate rural providers’ perspectives on challenges and recommendations for ACEs mitigation in their community from a cross-sector context.

**Participants and setting:**

Participants were 74 providers enrolled in an online ACEs training. Participants lived and/or worked in five rural Missouri counties with high drug overdose death rates.

**Methods:**

This was a conventional content analysis of written responses to structured discussion prompts from three training modules. Analysis included familiarization, inductive identification of major codes, and refinement of codes, followed by independent coding by two authors until consensus, and reporting major patterns. Artificial intelligence supported preliminary code development. An adapted Sequential Intercept Model (SIM) framework organized challenges and recommendations by setting.

**Results:**

Results included personal stories about how ACEs impacted their communities through overdose, suicide, homelessness, unemployment, and adverse health effects. Settings included schools, government, healthcare, daycares, criminal justice, Extension, fire departments, libraries, and nonprofits. Participants recommended expanding the ACEs definition to include satanic abuse, bullying, incest, war, genocide, military family stressors, and death of a loved one. Intervention recommendations included whole-family interventions, community-wide training, trauma-informed training, expanded access to treatment, classes in prison/jails, and fostering positive childhood experiences.

**Conclusion:**

Community voice is necessary to contextualize setting-specific insights that inform development of upstream interventions appropriate for and tailored to the community. Participants identified both strategies that could be incorporated into everyday practice—including empathy, listening, providing judgment-free and safe spaces, providing food for children experiencing hunger, encouraging positive childhood experiences, mentoring, and connecting individuals with resources for food, shelter, clothing, and mental health—and broader structural changes that may require policy-level action. The adapted SIM organizes these concepts.

## 1 Introduction

Mortality rates in the United States (U. S.) have been rising, especially for adults in rural areas, creating a rural mortality penalty (1). Significant contributors to these rising mortality rates include “deaths of despair,” or deaths from suicide and drug poisoning, including from opioids (2), with suicide death rates highest in rural areas (3). Deaths of despair were the fifth leading cause of mortality in the U. S. in 2021 and increased 2.5-fold between 1999 and 2021 (4). This trend was referred to as a crisis for working-age adults, with vulnerability identified as an upstream culprit (5). Indeed, a rising demand for relief from physical and emotional pain, coupled with the widespread availability of prescription opioids beginning in the late 1990s, contributed to the rise in overdose deaths (5). Several recent federal strategies, one on drug control (6) and one on suicide prevention (7), recommended addressing adverse childhood experiences (ACEs) as a root cause at the community level.

ACEs are a powerful upstream target for interventions to combat deaths of despair. In fact, the intersection of ACEs, suicide, and overdose has been a priority area for federal agencies such as the Centers for Disease Control and Prevention (8). Cumulative exposure to ACEs is associated with an increased risk of substance use (9, 10), overdose (11, 12), and suicide (13). In the U. S., 63.9% of adults report ≥ 1 ACE, and 17.3% report ≥ 4 (14). ACEs traditionally include physical abuse; emotional abuse; sexual abuse; physical neglect; emotional neglect; household member with a serious mental illness, with a substance use disorder, or incarcerated; or witness to intimate partner violence in the home (15). More recently, consideration for additional experiences has included community violence, discrimination, and economic hardship in childhood (16).

Compared to urban children, rural children had higher rates of exposure to parental separation/divorce (30.6% vs. 23.4%), parental death (5.0% vs. 3.1%), household incarceration (13.3% vs. 7.4%), household violence (8.0% vs. 5.1%), household mental illness (9.4% vs. 7.4%), household substance abuse (12.3% vs. 8.4%), and economic hardship (30.2% vs. 25.0%) based on results from the 2016 National Survey of Children’s Health (17). Similarly, results from the 2021–2022 National Health Interview survey found rural children were more likely to experience almost every type of ACE examined compared with their urban peers: victim or witness of neighborhood violence (6.8% vs. 4.8%), being separated from a caregiver due to incarceration (7.9% vs. 5.1%), living with a person with mental illness or severe depression (10.3% vs. 7.7%), living with a person with substance abuse issues (10.6% vs. 7.0%), experiencing verbal abuse from an adult they lived with (5.4% vs. 3.3%), and ever having their basic needs not met (4.2% vs. 2.6%) (18). Adjusted models showed no significant difference between urban and rural children experiencing ≥ 4 ACEs (17, 18).

There is a limited body of literature offering a contextual understanding of rural individuals’ perspectives on ACEs. Existing qualitative studies examine: screening and referrals in the health care setting (19–21); intervention and training in school settings (22, 23); parenting curriculum delivered in community settings (24); and perspectives of childhood sexual abuse among older adults living with HIV in clinical settings (25, 26). One additional article described a collaborative, community-led systems-mapping project facilitated by the Rural Opportunity Institute to understand lessons learned and recommendations for communities looking to apply systems thinking (27). Finally, our previous work is among this literature and includes a statewide sample of rural healthcare providers’ perspectives from an online ACEs training (28, 29).

Public Health 3.0 calls on us to engage multiple sectors and community partners to generate collective impact (30). To support this call, we evaluate provider perspectives from a broad range of sectors on how ACEs might be addressed within rural communities. Rural communities are known for being resource-limited, underscoring the importance of recognizing there are unique challenges with addressing health and well-being in these areas. The objective of this study is to evaluate rural providers’ perspectives on challenges and recommendations for ACEs mitigation in their community from a cross-sector context.

## 2 Materials and methods

### 2.1 Study design

This qualitative evaluation of discussion posts from an online training examines perspectives on ACEs from among cross-sector providers living or working in five rural Missouri counties.

### 2.2 Setting and participants

An online ACEs training “Adversity to Action” launched in May 2024. The training was available to individuals living and/or working in Missouri’s Crawford, Dent, Phelps, St. Francois, and Washington counties. These rural counties had the highest rates of drug overdose deaths in Missouri in 2021, followed only by St. Louis City (31). Additionally, they were among the top 20% of Missouri counties most vulnerable to opioid overdoses, as reported in the Missouri Department of Health and Senior Services’ 2022 vulnerability assessment (32). Within the five counties, providers from diverse sectors were invited to complete the training via email and snowball recruitment. The term “providers” is used broadly to include professionals in roles that intersect with individuals or families across communities. Recruitment included the following sectors: healthcare, behavioral health, housing, law enforcement, daycares, child and family services, corrections, local public health agencies, schools, libraries, emergency medical services (EMS), social services, and non-profit organizations.

### 2.3 Training development

The Adversity to Action training was available online at no cost through the University of Missouri Extension’s Canvas learning platform. The training was asynchronous and designed to take approximately 3–4 h to complete. This training was designed by JMK, and builds from a previously developed, implemented, and tested ACEs training that was delivered statewide to rural healthcare providers (28, 29). The content was modified to include the latest ACEs literature and tailored to individuals living and working in the five target counties. The evaluation of the current training, including pre/post knowledge assessments and translation to action, is described elsewhere (33).

The University of Missouri’s office of Continuing Education for Health Professions (CEHP) reviewed the Adversity to Action training and awarded it continuing education (CE) credit. Participants could claim up to 3 American Medical Association Physician’s Recognition Award Category 1 Credits.™ Additionally, CE was available for licensed professional counselors, social workers, and psychologists with Missouri licenses. EMS CE credit was available starting in October 2024. Participants could claim CE credit or a certificate of completion at no cost if they completed the following requirements: (1) a pre-training demographic survey; (2) all six discussions; (3) all five quizzes, earning a grade of >80% on each; and (4) a post-training evaluation survey.

### 2.4 Data sources

Data from the pre-training demographic survey and three of the six discussions were included in this analysis: (1) *What Are Adverse Childhood Experiences?* (2) *How Do Adverse Childhood Experiences Impact Health?* and (3) *What Can We Do to Address Adverse Childhood Experiences?* Participants were instructed to share only information they were comfortable sharing, and to not include identifiable information. Participants were able to view other participants’ posts. Discussion prompts are included in Table 1.

**Table 1:** Adversity to action training discussion prompts.

Training module and discussion prompts
What are adverse childhood experiences (ACEs)?
● Have you encountered individuals exposed to ACEs professionally or through personal relationships? Which category of ACEs did this include? Please explain. ● Are there types of childhood trauma not mentioned that you feel should be included in the definition of ACEs? Please explain. ● Please share any points you are comfortable sharing regarding suicide, drug use, or drug overdose deaths in the 5-county area of focus. What are you seeing? What thoughts or recommendations do you have?
How do adverse childhood experiences impact health?
● Do you have reason to believe a person’s childhood experiences can affect their long-term health or well-being? Please explain. ● Have you witnessed an association between a person’s number of ACE exposure types (abuse, neglect, and household dysfunction) and their trauma burden? Please explain. ● What are your thoughts on counting the number of ACEs exposures, versus the severity and/or frequency of trauma occurrences? Please explain.
What can we do to address adverse childhood experiences?
● What is your philosophy on how to implement trauma informed care? ● Professionally, how have you identified people with ACEs? Do you have a favorite screening tool? ● At what level is your organization in the trauma-informed change process? ● Are there opportunities for you to participate in helping transform your organization into a trauma-informed organization? Please describe. ● What actions have you taken to support people with ACEs, personally or professionally? What has worked in your experience? ● How do you encourage or reinforce positive childhood experiences (PCEs) for parents or children? ● Do you have stories of hope and resilience you can share? ● What unique barriers or challenges exist in your county? What solutions do you recommend?

### 2.5 Data analysis

The study sample includes participants who completed the pre-training demographic survey and at least one discussion prompt. Participant demographics were characterized with frequencies and percentages.

Qualitative content analysis interprets the content of text through the systematic classification process of coding and identifying patterns (34). Our qualitative analysis followed conventional content analysis of written responses to structured discussion prompts, using iterative, team-based consensus coding and an inductive approach (34). This approach allows categories to emerge from the data, with codes derived from the text that become the coding scheme.

First, we read the data to become familiar with its content. Then, preliminary codes were identified by us and separately by artificial intelligence (AI). Large language models, like ChatGPT, are increasingly supported for qualitative research given their ability to synthesize vast quantities of text (35, 36). AI has been used for qualitative data analysis for more than 25 years, and is useful for coding and generating accurate summaries (36). We pilot tested ChatGPT’s replicability by inputting a subset of anonymized data and identical prompts into each author’s ChatGPT accounts. The output was substantively similar. Next, we input an anonymized, limited dataset into ChatGPT from each of the three discussion posts, one at a time. We asked ChatGPT to perform the following steps with each iteration: familiarization by reading all text multiple times; open coding by identifying recurring ideas; and grouping related codes into broader patterns. Next, we reviewed ChatGPT’s outputs against their own and, drawing on their expertise, discussed revisions. Then, we independently applied the coding scheme to a sample set of data and met to discuss discrepancies and further revise the coding scheme. We then coded the remaining data independently, and met to discuss divergence and patterns until full agreement was reached. Discussion posts could receive multiple codes if the content addressed multiple content areas. Coding was conducted in Microsoft Excel, maintaining records of the analysis process. Finally, illustrative quotes were selected for each category. Quotes were lightly edited for spelling, length, and readability while maintaining content.

Trustworthiness considerations (37) were operationalized as follows. Transferability is described above with our sampling strategy, which was broadly inclusive of varying “provider” roles within the community. Regarding positionality, coding was developed in part based on author expertise areas (epidemiology, program evaluation, and behavioral neuroscience). Considering reflexivity and credibility, we operationalized confirmability by triangulating the initial coding scheme against each other’s recommendations as well as AI to strengthen accurate representation and objectivity. Also, we independently applied the coding scheme and discussed discrepancies. Saturation was not an objective given the nature of the secondary data from the online training.

A Sequential Intercept Model (SIM) is a framework used for community planning to map and assess interception points in how people move through various settings (38). SIM has been applied to the criminal justice system (39, 40), and an emergency department related to opioid overdose (41). Here, we adapted the SIM concept as an organizing framework of patterns following the conventional content analysis toward opportunities for ACEs-related prevention, referral, and intervention across community settings over the life course. Rather than representing an individual’s progression through the traditional SIM intercepts, the adapted model identifies settings in which cross-sector providers may encounter individuals affected by ACEs and opportunities to contribute to primary, secondary, or tertiary prevention. To develop the adapted model, we first grouped participant sectors into broader setting categories and then filtered discussion comments by setting to identify setting-specific observations, challenges, and recommendations.

### 2.6 Ethical considerations

This study was approved by the University of Missouri’s Institutional Review Board (project number 2129989) as an exempt secondary analysis.

## 3 Results

### 3.1 Enrollment and discussion prompt responses

Initial recruitment emails were sent to over 400 partners; however, the final recruitment denominator could not be determined because snowball recruitment was used. From May 2024 through February 2025, 127 individuals enrolled in the training. Of those 127 individuals, 99 (78.0%) completed the pre-training survey. Of those 99 individuals, 74 (74.7%) also completed at least one discussion prompt, for a final sample of *N* = 74. Participants posted a total of 203 discussion-prompt responses. On average, participants responded to 2.7 (SD = 0.6) prompts and posted 1.0 (SD = 0.1) response per prompt. Participation in each discussion prompt declined progressively throughout the training. The number of unique individuals and responses for each prompt were as follows: *What Are Adverse Childhood Experiences?* (74 individuals, 75 posts); *How Do Adverse Childhood Experiences Impact Health?* (67 individuals, 69 posts); and *What Can We Do to Address Adverse Childhood Experiences?* (57 individuals, 59 posts). Of the 74 participants included in the final sample, 18 (24.3%) did not complete the course requirements.

### 3.2 Participant characteristics

Table 2 shows the characteristics of the *N* = 74 participants included in the final sample. All participants lived or worked in the target counties. All 25 participants who lived in an “other” county worked in one of the five target counties or held a statewide position. Only 1 of the 8 participants who worked in “other county or statewide” worked in an “other” county, and this participant lived in one of the five target counties.

**Table 2:** Characteristics of adversity to action training participants who completed the pre-training survey and at least one discussion prompt (*N* = 74).

Variable	*n* (%)
Personal attributes	
Gender	
Female	63 (85.1%)
Male	9 (12.2%)
Age group	
18–24	3 (4.1%)
25–34	17 (23.0%)
35–44	26 (35.1%)
45–64	26 (35.1%)
65–84	1 (1.4%)
Race	
White	69 (93.2%)
Black or African American	3 (4.1%)
Ethnicity	
Hispanic or Latino	1 (1.4%)
Not Hispanic or Latino	68 (93.2%)
Grew up in a rural area	
Yes	56 (75.7%)
No	18 (24.3%)
In which Missouri county do you live?	
Crawford	2 (2.7%)
Dent	6 (8.1%)
Phelps	18 (24.3%)
St. Francois	13 (17.6%)
Washington	10 (13.5%)
Other	25 (33.8%)
Education level	
Regular high school diploma or GED	3 (4.1%)
Some college	8 (10.8%)
Associate degree	7 (9.5%)
Bachelor’s degree	26 (35.1%)
Master’s degree	24 (32.4%)
Professional, medical, or doctorate degree	6 (8.1%)
Employment attributes	
In which Missouri county/counties do you work?	
Crawford	1 (1.4%)
Dent	6 (8.2%)
Phelps	17 (23.3%)
St. Francois	9 (12.3%)
Washington	4 (5.5%)
More than 1 target county	28 (38.4%)
Other county or statewide	8 (11.0%)
Employment setting	
Child advocacy center	14 (18.9%)
School or school district	10 (13.5%)
Government	9 (12.2%)
Community-based organization	8 (10.8%)
Federally qualified health center or look-alike	8 (10.8%)
Local public health agency	6 (8.1%)
Other	19 (25.7%)
Profession	
Behavioral health specialist	12 (16.2%)
K-12 education: Teacher or administrator	10 (13.5%)
Director or leadership	6 (8.1%)
Other	46 (62.2%)

Sample sizes for individual demographic characteristics may not sum to the total sample size due to missing data. Denominators are as follows: gender (*n* = 72); age (*n* = 73); race (*n* = 72); ethnicity (*n* = 69); work county (*n* = 73).

Employment settings included child advocacy centers (18.9%), schools or school districts (13.5%), government (12.2%), community-based organizations (10.8%), federally qualified health centers or look-alikes (10.8%), local public health agencies (8.1%), and other settings, which included other health settings (5.4%), daycares (4.1%), criminal justice settings (4.1%), university Extension (2.7%), rural health clinics (2.7%), critical access hospitals (2.7%), fire departments (1.4%), libraries (1.4%), and nonprofits (1.4%). The most common professions were behavioral health specialists (16.2%), K-12 teachers or administrators (13.5%), and directors or other leadership roles (8.1%). Other professions that were indicated included social workers (6.8%), forensic interviewers (5.4%), prevention specialists (5.4%), nurses or nurse practitioners (2.7%), advocates (2.7%), community outreach coordinators (2.7%), EMS personnel (2.7%), and law enforcement officers (2.7%).

### 3.3 Summary of qualitative analysis

Patterns and illustrative quotes are reported in Table 3. Findings for *What are Adverse Childhood Experiences?* included: ACEs are perceived as common (widespread across the community and sectors; ACEs are varied and multiple; ACEs are embedded in the community); ACEs are perceived to have community-level impact (rising rates of substance use, overdose deaths and suicide; drug use, homelessness, and unemployment are co-occurring; residents know multiple individuals affected; ACEs compound social and economic impacts); substance use/misuse is perceived as common (drug use and addiction are perceived as common and increasing); participants describe intergenerational transmission (dysfunctional family behaviors perpetuate the cycle), participants provide recommendations for community-level change (more treatment centers and therapy services; emphasis on trauma-informed approaches rather than just medication; community-wide education and prevention efforts; address structural barriers such as transportation, broadband access, affordable housing, and cost; advocacy; listening and empathy are foundational), and suggest new types of ACEs to expand the traditional definition (satanic abuse, bullying, incest, war, genocide, military family stressors, death of a loved one).

**Table 3:** Qualitative analysis results by adversity to action module: patterns and exemplary quotes based on participants’ perceptions and self-report.

Training module and discussion results
What are adverse childhood experiences
A. ACEs are common (e.g., how often do participants come across people with ACEs) I have encountered many individuals who were exposed to ACEs, from divorce, parents with substance abuse, incarceration, and sexual, verbal, and physical abuse. (ID 17, local public health agency) The [student circumstances] that most frequently come to mind are parents struggling with substance abuse, food withholding, caregiver separation due to removal from the home, incarceration, etc. I would say caregiver substance abuse is the most frequent issue (ID 26, school or school district) Working on the ambulance I have encountered many people dealing with the effects of ACEs. I knew I may not be able to help them in their time of need, but having the ability to empathize with them made me a better advocate for their care. (ID 35, critical access hospital) Between our office and as a police officer, I encounter people daily who have experienced all three categories of ACES. Most of them deal with substance abuse or co-occurring issues where their decisions are based on and derived from past experiences. Past trauma often plays a major role in current conditions and lifestyle choices. (ID 40, criminal justice) Working at a Juvenile Detention Center, almost all of the youth I’ve met have a history of ACEs. It includes all types of abuse and neglect, witnessing domestic violence in the home, parental incarceration, divorce, and parental substance abuse. Drug use and overdose are affecting so many families, and it’s becoming harder for young people to avoid exposure to drugs. (ID 61, government) B. Community-level impact (e.g., ACEs are pervasive across communities) Suicide and drug abuse has been rapidly increasing in my county of late. (ID 1, local public health agency) Drug use, overdose, and homelessness are prevalent in my county. Most common are heroin and fentanyl. (ID 4, school or school district) My daughter is 23 and knows over 21 people her age that have died over the past 5 years from drug overdose or suicide in [redacted] counties. (ID 6, local public health agency) In our area of operation, we have seen an increase in drug use, overdose deaths, unemployment, and homelessness. (ID 40, criminal justice) I spent 30 years in the Fire Service. The use of drugs, alcohol, and death by suicide are higher than most people want either admit or see. (ID 47, child advocacy center) C. Substance use/abuse (e.g., how common is substance use, what have participants seen) Every day at work I see individuals affected by ACEs, [commonly] individuals whose parents have/had a substance use disorder, were divorced and the child experienced abuse/neglect. (ID 49, rural health clinic) I have seen more overdoses and drug use than I ever did growing up. A lot of children, mainly teenagers have grown up seeing their parent or friends’ parent on drugs. And they eventually give it a try. It only takes one time for you to get addicted or worse, lose your life. (ID 68, fire department) D. Intergenerational transmission (e.g., trauma passed from parents to children; perpetuation of trauma across generations affecting parenting and child development) My childhood household was dysfunctional; both parents were alcoholics and my mother dealt with mental illness. We were very poor and endured emotional trauma and neglect. (ID 2, community-based organization) Many participants that I work with have been abused physically or come from dysfunctional families. Many come from families with mental illnesses, that they now have as well or from parents that were drug addicts. (ID 3, community-based organization) Grandparents are doing their best to deal with the emotions/decisions of their kids along with the emotions/decisions of the grandchildren they are raising. The grandparents are often exhausted and worn down, causing emotional struggles. (ID 13, school or school district) E. Recommendations or a call to action (e.g., a need for a systemic response) The availability of resources, treatment centers, and affordable housing could assist with the decline of drugs in the area. (ID 3, community-based organization) Change needs to happen within homes and families. It is so discouraging to watch children be placed on psychotropic medications for symptoms that often times are behavioral responses to ACEs. Medications are a band-aid to emotional trauma, instead of working with the entire family unit to make changes…I see a lack of effective mental health services being offered, which I believe is partially due to a lack of service providers and large caseloads. (ID 16, school or school district) It would be incredibly beneficial to make a class similar to this one that can be taught in the prison/jail systems. The inmates often have nothing but time, and will engage in any kind of activity that gets them out of their jail cell. We should be educating people directly while also offering the support and treatment needed to work through these traumatic experiences. (ID 32, government) One of the best things we can do to even start to help people is to just listen. Everyone has a story, only some get a chance to tell it. The others often have no one to tell it to and the emotions get trapped inside causing long-lasting damage. (ID 40, criminal justice) Solving this issue has to start with recognition that we have a problem in rural counties of Missouri then developing a plan to answer the needs. (ID 47, child advocacy center) We need more prevention training, educating parents, professionals and the community. Transportation is a barrier. In some rural communities there is lack of broadband infrastructure or unreliable service. The cost of devices and service are also a direct barrier to purchase. (ID 57, other health setting) F. A new type of ACE that is not part of the traditional definition (e.g., bullying) We adopted two children who had received every type of abuse to include emotional, physical, sexual and one not mentioned: satanic. The latter was way harder for the kids to try and overcome. (ID 14, school or school district) School bullying has become a prevailing issue and kids are experiencing significant trauma linked to the peers they have to be around on a daily basis. (ID 20, federally qualified health center/look-alike) Incest is not called out for what it is, and there is not much education on it as people still feel the word is taboo…I do feel that incest opens up an entirely different category that I see entirely too much. (ID 32, government) The broader discussion about war and genocide also applies as I have worked with military base populations, or on reservations and with refugees. Also gang, organized crime experiences and systems of belief abuse would come into play. (ID 37, federally qualified health center/look-alike) Death could be added to the definition of ACEs. It affects their sense of safety and security, removes a person that was a support/part of their life, and causes emotional trauma. (ID 61, government)
How do adverse childhood experiences impact health
A. Mental or emotional health consequences (e.g., depression, anxiety, PTSD, emotion regulation) I think that we have children who are not actually ADHD, but more trauma related. (ID 25, daycare) I am morbidly obese, I smoked for years, I had zero self-esteem and depression since childhood. Now that I’m older, I can see some of those ACEs and where I might have missed them but nonetheless, they are there. (ID 41, other health setting) A child with ACEs has a higher risk of suicide attempts…ACEs can change the way you have relationships, and how you respond to things. So, without services and resources you could fall down a deep hole of emotional dysregulation as well as risky behaviors. (ID 55, child advocacy center) A youth’s father may be incarcerated, their mother uses drugs, and mother’s boyfriend is violent to mom in front of the youth. The youth is so overwhelmed with stress that their brain is on overload all the time that it presents with a mental health diagnosis or using drugs to cope. When the youth does not have drugs available to numb the pain, then they feel everything and can become suicidal. (ID 61, government) B. Physical health or chronic disease (e.g., heart disease, diabetes, inflammation, chronic stress) A lot of health issues are yet to be identified and linked to ACE scores such a repeated visits to the chiropractor for muscle issues, migraines, fibromyalgia, auto-immune diseases, etc. (ID 20, federally qualified health center/look alike) Some kids seek help and it can have a positive outcome, but it is more likely to lead them to risky behaviors that can impact their health (lung cancer, liver disease, heart disease, obesity) as well as their emotional and physical well-being. (ID 55, child advocacy center) Witnessing a person who was raped as a teen, put through what they called “electro-therapy” to make them forget the experience, marry an alcoholic, smoke, have nerve problems to the point they could not drive, have several health issues and end up with terminal cancer at age 58. (ID 64, daycare) C. Behavioral or lifestyle coping mechanisms (e.g., substance use, poor diet, risk taking) If a child does not get their basic needs met or the foundation of feeling loved and connection are not met then eventually the child will look for ways to suit their needs. This usually looks like getting into a toxic relationship, using drugs/alcohol, over-eating, etc. All in attempts to fill a void from childhood traumatic experiences. (ID 33, federally qualified health center/look alike) I have an ACE score of 10. I have had to unlearn behaviors over the years from my own upbringing to avoid placing an unnecessary burden on my spouse and children. (ID 54, child advocacy center) I have seen children that have experienced parental drug use with multiple arrests and incarcerations. …They fall behind in school, get in trouble in school, and often end up dropping out of school. They then begin drug use themselves at an early age continuing that cycle. (ID 56, child advocacy center) I recently lost my adult daughter to a fentanyl overdose. Her ACE score was high and she suffered through years of drug and alcohol abuse/addiction, trafficking, abusive relationships as well as time in and out of jail and prison as an adult. (ID 66, child advocacy center) D. Family impact or intergenerational impact (e.g., how ACEs affect parenting, cycles of trauma from parent to child) My household was dysfunctional; both parents were alcoholics, and my mother dealt with mental illnesses her entire life. Four out of the seven siblings have either high blood pressure or diabetes. (ID 2, community-based organization) I have family members who grew up in poverty and their parent had a substance use disorder. Most of [them] have health issues and substance use disorder themselves. (ID 21, other health setting) Growing up around smoking or violent behavior where this is normal and accepted in the adults a child looks up to create a sense of normalcy for these behaviors and an expectation that these behaviors are acceptable. (ID 24, university Extension) I have been in law enforcement for years, and have seen teens follow the footsteps of their parents. I have even seen a daughter and mother share a jail cell. That same mother and daughter was the perfect example of their “trauma burden” showing their repetitive drug usage and addictive behaviors in life that were unbroken due to lack of ability to pay for and attend treatment services. (ID 32, government) E. Recommendations or interventions (e.g., healing, prevention, resilience) I think if there was a way for the person to score the severity of the incident it would be helpful. For example, a death of a parent may be extremely difficult for one individual but maybe not for another. (ID 10, school or school district) One can overcome the trauma and live a productive life. But it takes counseling, working with mental health providers, and building up a support team. (ID 30, federally qualified health center/look alike) The total count of these exposures needs to be documented for their healthcare providers. [This] allows providers a chance to counter these bad behaviors before they occur. (ID 35, critical access hospital) If we had access to services that are available today, [my daughter’s] outcome may have been different. Helping children heal and giving them the tools they need is one of the most important things we can do. (ID 66, child advocacy center) Counting the ACEs may help [identify] just how bad the person needs help. Heck most of the time just giving the child the attention they so badly crave from their own family can help that child understand that life is not always so bad. I believe everyone should treat each other with respect and love, especially since we do not know what they are going home to. (ID 68, fire department)
What can we do to address adverse childhood experiences
A. Create safe and supportive environments (e.g., listening, showing compassion, everyday acts of care, mentoring, building trust, promoting positive childhood experiences) Listen, hear their story; once they can see someone cares, that trauma can be worked on, it does not ever change the past but it can give them a better thought for the future. (ID 9, federally qualified health center/look alike) As a teacher, I believe the biggest difference that I can make is to build positive relationships and rapport. (ID 26, school or school district) We provide a safe and engaging place for families to take their children at no cost. (ID 28, library) As law enforcement, we go to several calls time and time again. Repeat offenders, repeat calls, repeat crisis. We become a familiar face in the moment of crisis. It only takes a moment of compassion to slow down and show the person in crisis that you care. (ID 32, government) By successfully offering [young people] options and opportunities for them to abstain from drugs and alcohol, I can help create an environment that fosters positive childhood experiences. (ID 48, community-based organization) As a therapist and an advocate, I am constantly trying to highlight and encourage the PCEs of parents and children. They are often emotionally overwhelmed. Helping them identify things they are doing well/right has been helpful for the families I have served. (ID 51, child advocacy center) B. Provide resources to children, adults, families or make resource connections We provide information and resources to educate youth, families and communities on the dangers of underaged substance, tobacco and alcohol use. I have taken the ASSET Builders course, learning tools to help families be more resilient with support, empowerment, constructive use of time, build positive values and social competencies. We also facilitate a mentoring program for at-risk youth. (ID 2, community-based organization) When we have children show signs of ACEs, we monitor them closely, have transparent conversations with families to help identify the cause, then work to develop a care plan for the child and family. We provide a list of community resources and help them get in contact. We ensure that our staff understand to make connections with the children so that they find safety in our staff and build trusting relationships to help them. (ID 25, daycare) Law enforcement and first responders can play a role in making those connections and following up to make sure the person takes advantage of those resources. We cannot force anyone to get help, but I do believe having caring support that checks in and follows up makes a difference. (ID 32, government) C. Organizational education, training, and change (e.g., staff development, organizational-level policy shifts, trauma-informed care implementation) More research and funding into therapeutic programs that work for ACEs needs to be [available]. Missouri has a shortage of therapists. (ID 20, federally qualified health center/look alike) One of the best ways to implement trauma-informed care is to require ongoing training of the staff and reevaluate procedures and policies on a regular basis. The substance use field is full of stigma and bias. It is important for staff members to treat people who have experienced trauma with dignity and respect. Training and evaluation keep the philosophy in the forefront. (ID 21, other health setting) As a specialized court program focusing on wholistic care, we want to implement every area of care, including trauma, in services provided. We started trauma-based Moral Reconation Therapy and ask our counselors to look at childhood trauma as a potential root to substance abuse. (ID 36, government) It is important that those within the various levels of the judicial system [are] trauma informed. This would provide a better insight to potential needs when determining a course of action that will reduce recidivism instead of enabling it. (ID 40, criminal justice) Most [child advocacy centers] now follow the “one interview” protocol. The child is recorded during the forensic interview and that way they do not need to repeat and re-live their trauma by sharing the story more than once. (ID 58, child advocacy center) D. Address structural gaps and systemic barriers (e.g., poverty, stigma, lack of funding, transportation issues, mental health workforce shortages) There is a lot of poverty, a lot of people do not have health insurance. Also that they may not know about [ACEs], they are conditioned to think that is normal. (ID 17, local public health agency) There is lack of family therapy that takes Medicaid or state insurance. I recommend that Medicaid be able to pay for family therapy. (ID 30, federally qualified health center/look alike) We lack mentoring or programs available to our kids. The kids in sports or activities usually have the means. It would be great for community programs and churches to lift up caring adults to cover the gap (like transportation) to help kids get involved in activities that are protective. (ID 67, government) E. Stories of hope and resilience or narratives of transformation (e.g., resilience, supportive adults, positive environments) One girl [in 5th grade] did not have any qualms about throwing a punch…and did poorly in academics. She was pulled from the home due to drugs, overdoses, and death. The foster home she went to was a success story. Her appearance was clean, her grades went from low F’s to high D’s, she spent less time in detention, and she looked happy and healthy. We gave her the “most changed” award for all of 5th grade. She cried. I now see her participating in community activities, like cheerleading, and am so thankful for the foster family. (ID 4, school or school district) I have seen a child grow up [into] a middle-aged adult. [He had] divorced parents, multiple stepparents, parental drug use, frequent parental incarcerations, and early drug use himself. He did have PCEs with other family members and completed therapy over the course of years. He has a family of his own [now] and is well-respected. His children have not experienced ACEs. That cycle of ACEs ended with him. (ID 56, child advocacy center) A young teenager that was emancipated at age 15 having a child at 16 after being raised by an alcoholic father and a mother [with] many ACEs. She dropped out of school, worked at restaurants and bars for a few years to raise her child on her own. She finally settled down with a decent man, got her GED, [then] Associate’s degree while raising 4 children. She worked in the public school system > 10 years, then went to work in a childcare facility and advanced to director and has been there for almost 20 years. (ID 64, daycare)

Findings for *How Do Adverse Childhood Experience Impact Health?* included: perceptions of mental or emotional health consequences (chronic stress, emotional regulation, ADHD, self-esteem, depression, suicide attempts), physical health or chronic disease (muscle pain, migraines, fibromyalgia, auto-immune disease, liver disease, heart disease, obesity, cancer); behavioral or lifestyle coping mechanisms (toxic relationships, substance use, over-eating, criminal activity, school absences); intergenerational impact (resulting chronic conditions and substance use, family norms, poor role modeling); and recommendations (modifying the ACEs definition to include severity of the exposure; counseling, screening in a healthcare setting, resource connections, skill-building, paying attention, being respectful).

Findings for *What Can We Do to Address Adverse Childhood Experiences?* included: to create a safe and supportive environment [listen, provide empathy, encourage, reinforce positive childhood experiences (PCEs), build rapport, compassion, opportunities to abstain from substance use]; provide or connect to resources (education, training on resilience, mentoring programs, following up on connections); organizational education, training, and change (implementing trauma-informed care; access to therapeutic programs; routinely educate staff on procedures and policies; a single interview to understand a story rather than re-traumatize with multiple interviews); address structural gaps (poverty; health insurance; expand Medicaid to include family therapy; transportation; free trauma therapy; mentoring); and stories of hope or resilience.

### 3.4 Sequential intercept model

Our adapted life-course intercept model informed by the SIM framework included setting findings of childhood (which included potential points of intercept through daycares, schools/school districts, and child advocacy centers); clinical (hospitals, rural health clinics, federally qualified health centers, and local public health agencies), community resources (libraries, government, fire department, Extension, and community-based organizations), and justice system settings (criminal justice). The discussion *How Do Adverse Childhood Experiences Impact Health?* did not contain any setting-specific content. Findings are presented in Figure 1.

**Figure 1:**
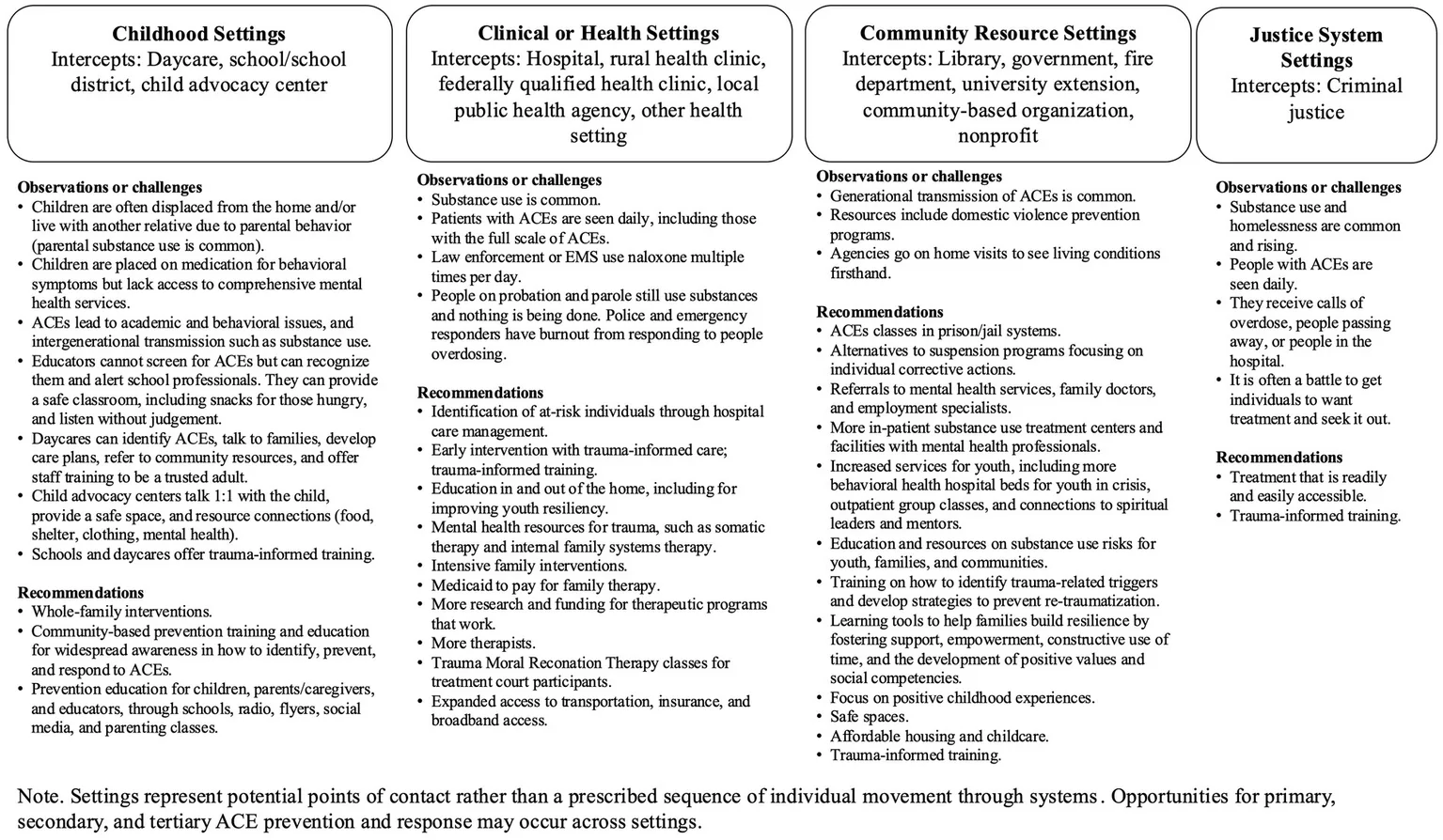
Adapted life-course intercept framework of adversity to action participant’s observations and recommendations, by setting.

## 4 Discussion

This study contributes to a paucity of literature on ACEs in rural settings by offering a contextualized understanding of a cross-sector of provider perspectives from five communities, selected for having among the highest rates of drug overdose deaths in Missouri. Participants report perceptions of widespread exposure to ACEs and associated community impacts, including pervasive substance use (including heroin and fentanyl); rising substance use, overdose deaths and suicide; homelessness and unemployment; and the intergenerational transmission of ACEs, described through anecdotes such as a daughter and mother sharing a jail cell. The reported community impact of ACEs is substantial, with one participant stating, “My daughter is 23 and knows over 21 people her age that have died over the past five years from drug overdose or suicide”.

Participants recommended expanding the ACEs definition to include satanic abuse, bullying, incest, war, genocide, military family stressors, and death of a loved one. Future research might explore the prevalence of each of these experiences, whether they are distinct from existing ACEs, and the association of these experiences with adult health outcomes. For example, conceptually, incest may fall within the definition of sexual abuse, however, the psychological implication of abuse experienced from a biological, compared to non-biological, relation may be distinct (see the participant’s response in Table 3).

This study uniquely responds to Public Health 3.0’s call to engage multiple sectors and community partners to generate collective impact, and to learn from the field for actionable data (30). Participants’ perspectives provide compelling insight supporting the need for action in these communities. Reducing ACEs has the potential to substantially lessen their health and economic burden on communities — the total annual costs attributable to ACEs are estimated to be $748 billion in North America, with more than 75% of these costs from individuals with two or more ACEs (42). A 10% reduction in ACE prevalence could equate to annual savings of $105 billion (42). In theory, these savings would be recovered by addressing the wide-ranging sequelae reported by participants, including chronic stress, low self-esteem, depression, suicide attempts, migraines, heart disease, obesity, cancer, toxic relationships, substance use, criminal activity, and intergenerational transmission of ACEs.

Community-level impact calls for community-level intervention. Recommendations in the SIM map can be used to plan and develop effective future umbrella and multisector interventions across the five counties, and could use RE-AIM as a planning framework for developing and evaluating interventions informed by these findings (43). Such an approach may be particularly valuable for engaging multi-sector partners and creating a systems-level community response to ACEs. Participants identified both strategies that could be incorporated into everyday practice—including empathy, listening, providing judgment-free and safe spaces, providing food for children experiencing hunger, encouraging positive childhood experiences, mentoring, and connecting individuals with resources for food, shelter, clothing, and mental health—and broader structural changes that may require policy-level action, including addressing poverty and barriers to health insurance access, broadband, transportation, family therapy coverage, and affordable housing.

Despite these enormous challenges, our providers expressed resilience in their own perspectives and actions, and offered resourceful strategies that included trauma-informed care across childhood, clinic, community-resource, and criminal justice settings. Stories of hope demonstrated transformation is possible.

### 4.1 Limitations

This qualitative evaluation examined how cross-sector rural providers interpreted and contextualized ACE-related concepts following participation in an online ACEs training. First, because participants responded to ACE-related educational content, responses may reflect educational priming, demand characteristics, or social desirability. Findings therefore represent participants’ reflections within the context of the training and should not be interpreted as independent measures of their pre-training perspectives. Second, although exposure to other participants’ responses may have influenced subsequent comments and reduced response independence, this type of participant influence is also inherent in interactive qualitative contexts such as focus groups. Next, the sample consisted of providers recruited through snowball sampling; therefore, findings reflect the perspectives of participating providers and may not be generalizable to the broader community. Additionally, participant attrition occurred as they advanced through the training, which may have limited the scope of later discussion posts. However, we anticipate this impact is minimal as 57/74 or 77% of participants who posted in the first discussion posted in all three. Findings from these providers may not be generalizable to other communities, as rural counties are often heterogenous and variable. Still, challenges identified are consistent with well-known rural challenges, including lack of transportation, broadband access, and mental health and healthcare provider resources. Finally, selection bias may have occurred in that those interested in ACEs may have opted to participate, while others may not have participated due to no interest in or awareness of ACEs or, conversely, concerns about being retraumatized if the participant had personally experienced ACEs.

### 4.2 Implications and future research

Findings represent a contextualized analysis of cross-sector provider perspectives. Future research should consider participants’ suggestions for an expanded definition of ACEs, to include bullying, satanic abuse, incest, war, genocide, military family stressors, and death of a loved one. Next steps include community-based dissemination of findings to educate providers on cross-sector challenges and strategies, and empower community-wide partnerships, interest groups, and mobilization. Findings related to structural challenges should be distributed to local legislators and community leaders.

### 4.3 Conclusion

This study provides three major contributions to the literature. First, it adds to a limited literature on ACEs in rural settings. Second, it uniquely supports the Public Health 3.0 initiative through capture of a broad cross-sector of community perspectives in order to inform localized, population-level interventions. Finally, it provides an adapted SIM framework to serve as a useful tool to guide actionable discussion with legislators and other community partners for macro-level intervention development.

## Data Availability

The datasets presented in this article are not readily available because of risk of re-identification. Requests to access the datasets should be directed to kappj@health.missouri.edu.
